# A Plasmonic Temperature-Sensing Structure Based on Dual Laterally Side-Coupled Hexagonal Cavities

**DOI:** 10.3390/s16050706

**Published:** 2016-05-17

**Authors:** Yiyuan Xie, Yexiong Huang, Weihua Xu, Weilun Zhao, Chao He

**Affiliations:** School of Electronic and Information Engineering, Southwest University, Chongqing 400715, China; hyxiong@email.swu.edu.cn (Y.H.); xiaohua2013@swu.edu.cn (W.X.); weilun@swu.edu.cn (W.Z.); hc2014@swu.edu.cn (C.H.)

**Keywords:** finite-difference time-domain (FDTD) method, dual hexagonal cavities, metal-insulator-metal (MIM) waveguide, plasmonic temperature sensor

## Abstract

A plasmonic temperature-sensing structure, based on a metal-insulator-metal (MIM) waveguide with dual side-coupled hexagonal cavities, is proposed and numerically investigated by using the finite-difference time-domain (FDTD) method in this paper. The numerical simulation results show that a resonance dip appears in the transmission spectrum. Moreover, the full width of half maximum (FWHM) of the resonance dip can be narrowed down, and the extinction ratio can reach a maximum value by tuning the coupling distance between the waveguide and two cavities. Based on a linear relationship between the resonance dip and environment temperature, the temperature-sensing characteristics are discussed. The temperature sensitivity is influenced by the side length and the coupling distance. Furthermore, for the first time, two concepts—optical spectrum interference (OSI) and misjudge rate (MR)—are introduced to study the temperature-sensing resolution based on spectral interrogation. This work has some significance in the design of nanoscale optical sensors with high temperature sensitivity and a high sensing resolution.

## 1. Introduction

A temperature sensor is an important kind of sensor used for temperature measurement in various applications. Compared with conventional electrical temperature sensors, the optical temperature sensors have more advantages, such as electromagnetic interference immunity, great sensitivity, large temperature range, fast response, and stability [1,2]. To date, many research efforts on optical temperature sensors have mainly focused on fiber-optic temperature-sensing technology. Accordingly, a variety of fiber-optic temperature-sensing methods have been investigated theoretically and numerically, including the fiber-optic sensors for temperature based on Bragg gratings [3,4], interferometers [5,6,7,8], surface plasmon resonance [9,10], and multimode interference [11,12]. However, these sensors are not suitable for chip-scale temperature sensors. To solve this problem of integration, new temperature-sensing technologies have been proposed and analyzed theoretically and experimentally, such as silicon photonic temperature sensor [13,14] and temperature sensors based on photonic crystal surface plasmon waveguide [15]. Nevertheless, their temperature sensitivities still need to be improved. Surface plasmons (SPs) have important applications in sensing. In the past, in order to confine surface waves at microwave and terahertz frequencies for compact sensing applications, a series of spoof localized surface plasmon (LSP) structures have been investigated for compact sensing applications, but they have complex structures and centimeter-scale sizes [16,17,18,19]. In recent years, surface plasmon polaritons (SPPs) have been considerably attractive for the realization of the subwavelength optical devices and circuits with high integration due to its specific property of overcoming the optical diffraction limit to the conventional photonic devices [20,21,22,23,24,25]. Due to its relatively easy fabrication and strong confinement to light in its structure, and relatively long propagation length for its low bend loss, metal–insulator–metal (MIM) waveguide has been used to guide SPPs waves at subwavelength scales as a potential candidate [26,27,28,29,30,31,32]. A variety of SPPs devices based on MIM waveguides has been investigated [26,27,28,29,30,31,32,33,34,35,36,37,38,39]. Particularly, among these devices, plasmonic sensors have received a lot of attention, owing to its unique advantage about the extremely sensitive response of the transmission spectrum to the change of the external refractive index [36,37,38,39]. Furthermore, according to the physical relation between the refractive index of dielectric medium and the temperature, the corresponding plasmonic temperature sensors with high integration and sensitivity have recently been studied theoretically and numerically [40,41,42,43]. However, few researches have focused on the parameter of sensing resolution, which is an important parameter in evaluating the minimum detectable change of environment temperature. Moreover, the interference between two adjacent spectra is rarely studied.

In this paper, we propose a plasmonic structure to construct a temperature sensor based on an MIM waveguide side-coupled with dual symmetric hexagonal cavities. Its transmission characteristics and temperature-sensing characteristics are investigated numerically by utilizing the finite-difference time-domain (FDTD) method. By tuning the coupled distance, the full width of half maximum (FWHM) of the resonance dip can be narrowed down with a maximum extinction ratio. Most importantly, we study the temperature-sensing resolution of the sensor based on two new concepts—optical spectrum interference (OSI) and misjudge rate (MR)—introduced in this paper. It indicates that the reduction of the FWHM of the transmission spectrum can improve the sensing resolution.

## 2. The Sensing Structure and Theoretical Analysis

**Figure 1:**
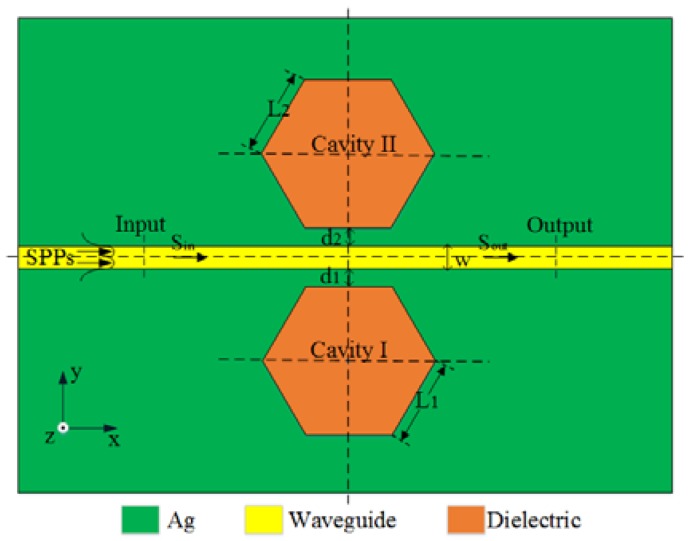
The Schematic of the plasmonic temperature-sensing structure with dual laterally side-coupled hexagonal cavities.

As can be seen in Figure 1, the two-dimensional (2-D) schematic diagram of the plasmonic sensing structure is depicted, which consists of an MIM waveguide with width *w* and two laterally coupled hexagonal cavities with side lengths *L*_1_ and *L*_2_ at a symmetric position. *d*_1_ and *d*_2_ are the coupling distances between the MIM waveguide and the cavities, respectively. S_in_ and S_out_ represent the amplitudes of the SPPs at the incoming and outgoing ports, respectively. The medium set in MIM waveguide is air with its refractive index of *n* = 1. The dielectric material with refractive index n_d_ is filled in two hexagonal cavities. Silver is selected as the metal, and its permittivity can be given by Lorentz-Drude model [44,45]:

(1) $$\varepsilon_{m}(\omega )=\varepsilon -\sum_{i=1}^{6}\frac{\omega_{p,i}^{2}}{\left(\omega_{o,i}^{2}-\omega^{2}+i\omega \omega_{d,i}\right)}$$

where *ε* represents the relative permittivity at finite frequency. *ω* denotes the optical angular frequency. *ω*_o_ is the frequency at resonance. *ω*_d_ and *ω*_p_ are the damping frequency and the plasma frequency, respectively.

The dispersion relation of the SPPs propagated in the MIM waveguide can be given by the equations as follows [28]:

(2) $$(\varepsilon_{m}k_{d})\tanh(\frac{wk_{d}}{2})+\varepsilon_{d}k_{m}=0$$

(3) $$k_{d}=\sqrt{\beta^{2}-\varepsilon_{d}{k_{0}}^{2}}$$

(4) $$k_{m}=\sqrt{\beta^{2}-\varepsilon_{m}{k_{0}}^{2}}$$

(5) $$n_{eff}=\beta /k_{0}$$

where *ε_m_* and *ε_d_* are the relative permittivities of the metal and the dielectric. *k_m_* and *k_d_* denote the wave vectors in the metal and dielectric. *β* is the propagation constant of the SPPs in the MIM waveguide. *k*_0_ is the wave number of the light in vacuum. *n_eff_* denotes the effective refractive index of the waveguide for SPPs.

If the resonance condition of the cavity is satisfied, the SPPs excited in the MIM waveguide would be coupled into the resonant cavity located next to the waveguide and form a standing wave. For a single plasmonic hexagonal cavity, the resonance wavelength *λ_m_* can be obtained theoretically by [46]:

(6) λm=(6L) × Re(Neff)m−ϕ/2π

where Re(*N_eff_*) is the real part of the effective index *N_eff_* of the hexagonal cavity, which is related with the refractive index of the dielectric medium. The positive integer *m* represents the order of the standing SPPs wave in the cavity. *φ* is the total phase shift for SPPs at all the corners of the hexagonal cavity. In this paper, we set *L*_1_ = *L*_2_ = *L* to make their resonance wavelengths at the same wavelength, and *d*_1_ = *d*_2_ = *d* to make the transmission characteristic of the sensing structure, which can be simplified by [47]:

(7) $$T(\omega )=\frac{{\left(\omega -\omega_{o}\right)}^{2}+{\left(1/\tau_{i}\right)}^{2}}{{\left(\omega -\omega_{o}\right)}^{2}+{\left(1/\tau_{i}+2/\tau_{w}\right)}^{2}}$$

where 1/*τ_i_* and 1/*τ_w_* denote the decay rate of the energy due to the internal loss of the cavity and the energy coupled into the waveguide from the cavity, respectively.

The refractive index *n* of the medium material has a relation with the ambient temperature *T* as follows [43]:

(8) $$n=n_{0}+dn/dT\;\times \;(T-T_{0})$$

where *T*_0_ is the reference temperature, and *n*_0_ is the corresponding refractive index of the medium material. *dn/dT* is the temperature coefficient of refractive index. A change in the temperature results in a change in the refractive index of the sensing medium. Temperature sensitivity (TS) can be defined as follows [43]:

(9) TS=dλmdT=6Lm−ϕ/2π × d[Re(Neff)]dT

Based on the above analysis, it indicates that we can construct a plasmonic temperature sensor based on the proposed structure.

Temperature-sensing resolution is an important parameter to evaluate the minimum detectable temperature change. The area of the overlap between two transmission spectra can be used to evaluate the sensing resolution. If an obtained transmission spectrum overlaps severely with the compared transmission spectrum, it becomes hard to distinguish the shift. In this paper, two new concepts are introduced to investigate the temperature-sensing resolution of the temperature sensor. At first, similar to the concept of intersymbol interference (ISI) in the communication field, OSI is introduced to describe the interference degree between two adjacent spectra. For the Gaussian wave, OSI can be defined as:

(10) $$\varphi =e^{-\frac{1}{2\alpha^{2}}}$$

where *α* denotes the relative root-mean-square width of the Gaussian wave, and is defined as:

(11) $$\alpha =\frac{\sigma }{\Delta \lambda }$$

where $\sigma =\tau_{FWHM}/\sqrt{2In2}$ represents the root-mean-square width of the Gaussian wave. *λ*_FWHM_ and Δ*λ* are the FWHM and the sensing space between two spectra.

Furthermore, we introduce another parameter of MR to further describe the accuracy of the shift detection. Misjudge rate *P*_MR_ is defined by:

(12) $$\begin{array}{cc} P_{\text{MR}} & =\frac{2\int_{-\infty }^{-\Delta \lambda /2}e^{-\frac{\lambda^{2}}{2\sigma^{2}}}d\lambda }{\int_{-\infty }^{\infty }e^{-\frac{\lambda^{2}}{2\sigma^{2}}}d\lambda }\\ & =2\Phi (-\frac{\Delta \lambda }{2\sigma })\;=2\left[1-\Phi (\frac{\Delta \lambda }{2\sigma })\right] \end{array}$$

**Figure 2:**
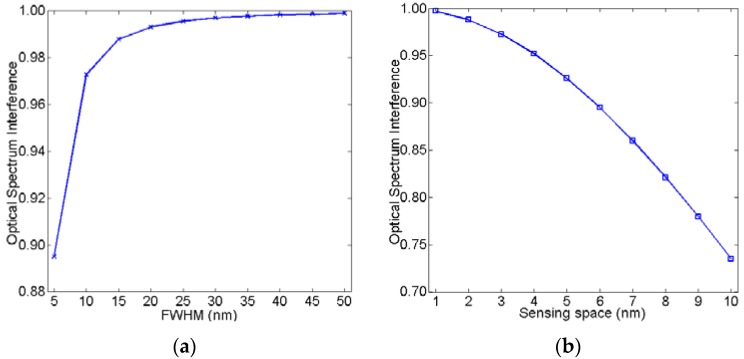
Optical spectrum interference as a function of (**a**) *λ_FWHM_* and (**b**) Δ*λ*, respectively.

**Figure 3:**
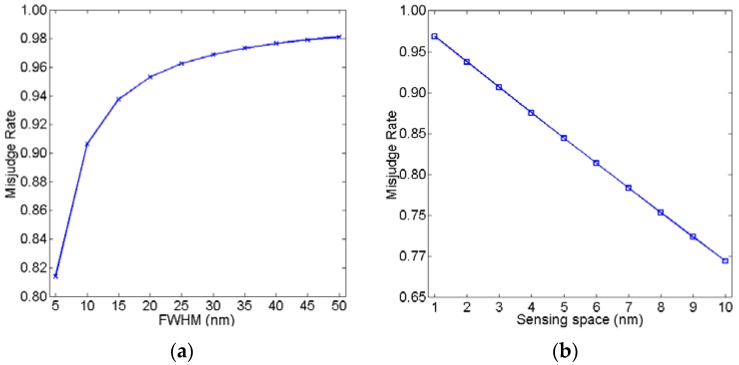
Misjudge rate as a function of (**a**) *λ_FWHM_* and (**b**) Δ*λ*, respectively.

According to Equations (10)–(12), OSI and MR are mainly dependent on parameters *λ_FWHM_* and Δ*λ*. Figure 2a shows OSI as a function with *λ_FWHM_*, assuming that Δλ = 1 nm. Figure 2b shows OSI as function with Δλ, assuming that *λ_FWHM_* = 30 nm. Similar to OSI, Figure 3a shows the relationship between MR and *λ_FWHM_*, assuming that Δλ = 1 nm. Figure 3b shows the relationship between MR and Δ*λ*, assuming that *λ_FWHM_* = 30 nm. As can be observed in Figure 2 and Figure 3, OSI and MR increase as FWHM increases. OSI and MR decrease as the sensing space increases. If OSI nears 1, it means that the two waves overlap severely. If MR nears 1, it is difficult to accurately detect the shift in the peak of the wave. Therefore, smaller OSI and MR are needed for a better sensing resolution.

## 3. Results and Discussion

**Figure 4:**
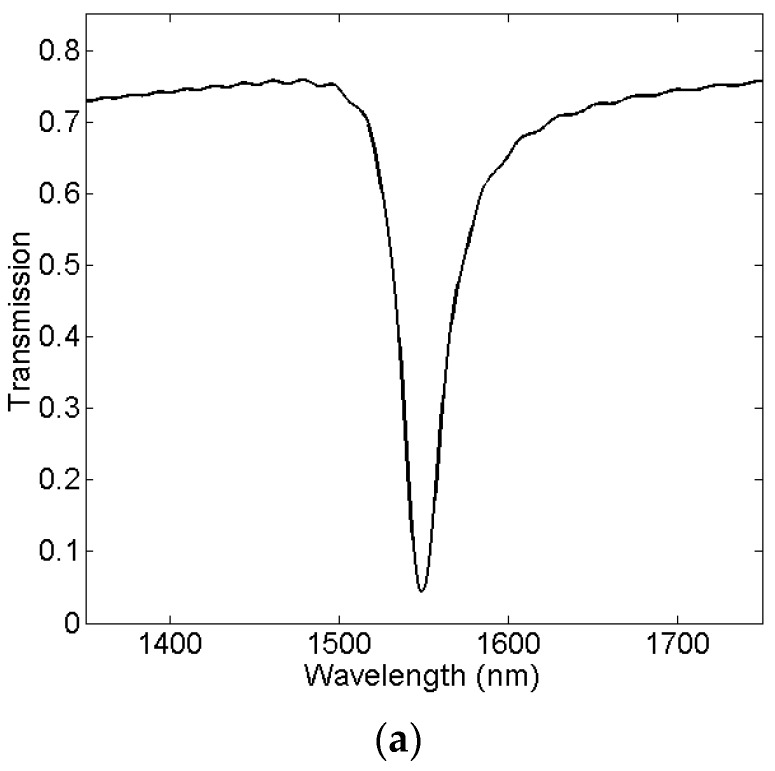

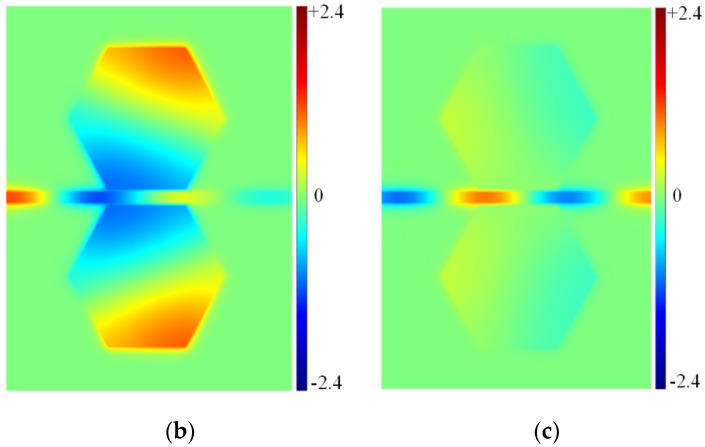
(**a**) Simulated transmission spectrum of the proposed sensing structure. The contour profiles of the field distributions of |Hz| at wavelengths of (**b**) 1548.5 nm and (**c**) 1500 nm, respectively.

The transmission characteristics and the sensing characteristics of our proposed plasmonic structure are investigated by the FDTD method, which is performed with the commercial EastFDTD software [48,49]. In the simulation, the spatial steps are set to be Δx = Δy = 5 nm, and the value of temporal step is set to be 0.5. Periodic boundary condition is applied in z-direction, and the perfectly matched layer boundary condition is applied in the x-direction and y-direction. Figure 4a displays the simulation result of the transmission spectrum, and Figure 4b,c show the field distributions of |Hz| at the incident wavelengths of 1548.5 nm and 1500 nm, respectively. The structural parameters of the plasmonic sensor are set to be *L*_1_ = *L*_2_ = *L* = 453 nm, *d*_1_ = *d_2_* = *d* = 20 nm, *w* = 50 nm, and *n_d_* = 1.0, respectively. As can be observed in Figure 4a, the minimum value, namely, resonance dip in the transmission spectrum occurs at a wavelength of 1548.5 nm. The resonance dip have an extinction ratio value of about 12.3 dB and a FWHM value of about 29.5 nm, respectively. In order to verify this result, we discuss their field distributions. As can be observed in Figure 4b,c, the energy of the SPPs in the waveguide is mostly coupled to the hexagonal cavities, and few energy is propagated to the outgoing port of the MIM waveguide at the resonance wavelength of 1548.5 nm. On the contrary, at the non-resonance wavelength of 1500 nm, almost no energy in the waveguide is coupled to the hexagonal cavities, but most propagate directly to the outgoing port of the MIM waveguide. 

**Figure 5:**
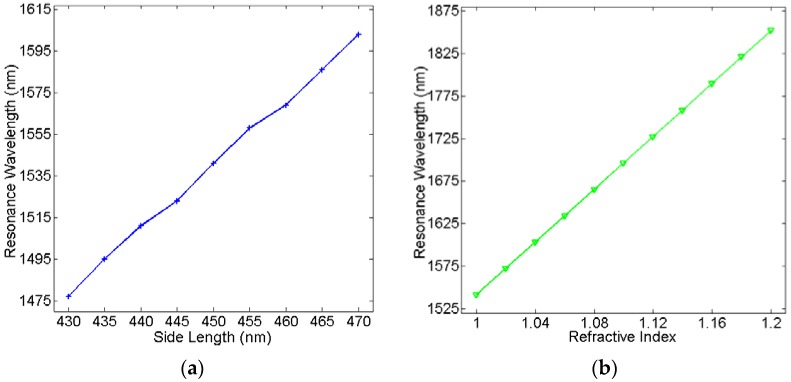
Resonance wavelength of the plasmonic sensor versus (**a**) side length *L* and (**b**) the refractive index *n_d_* of the dielectric material in the cavities.

Successively, we validate the resonance wavelength of the two cavities as a function with their side lengths and with the refractive index *n_d_* of the dielectric material in two cavities, respectively. As can be observed in Figure 5a, it plots a near-linear relationship between the resonance wavelength with the side length. And as shown in Figure 5b, the resonance wavelength has a completely linear relationship with the refractive index. These results can be explained by Equation (6). Therefore, the resonance wavelength can be changed by tuning the side length and the refractive index of the sensing material in the cavity can be calculated by measuring the resonance wavelength.

**Figure 6:**
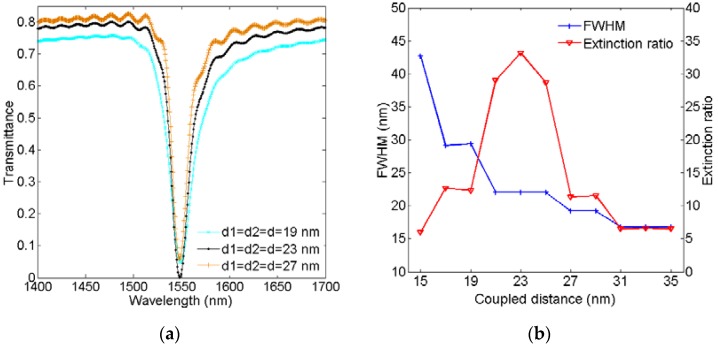
(**a**) Transmission spectra for *d* = 19 nm, 23 nm, and 27 nm; (**b**) FWHM and extinction ratio as a function of coupled distance *d*.

To investigate the influence of the coupling distance *d* on the transmission spectra of the plasmonic sensor, we set the coupling distance *d* increasing from 15 nm to 35 nm in increments of 2 nm, and other structural parameters are set to be *L* = 453 nm, *w* = 50 nm, and *n_d_* = 1.0, respectively. Figure 6a displays the different transmission spectra at the coupling distance *d* = 19 nm, 23 nm, and 27 nm, respectively. As shown in Figure 6a, the linewidth and depth of the resonance dip of the transmission spectrum can be affected by changing the coupling distance. Figure 6b shows the FWHM and extinction ratio of the resonance dip as a function of the coupling distance. As shown in Figure 6b, the FWHM of the resonance dip decreases as the coupling distance increases, and it reaches the minimum value of 16.8 nm after *d* = 31 nm, which is smaller than the result given in [47]. Unlike the FWHM, the extinction ratio of the resonance dip increases first and then decreases, and it reaches the maximum with a value of about 33 dB at *d* = 23 nm. Therefore, there is a trade-off between the FWHM and extinction ratio. Based on the above analysis, the FWHM of the resonance dip can be narrowed down, and the extinction ratio can reach a maximum value by tuning the coupling distance between the waveguide and hexagonal cavities.

**Figure 7:**
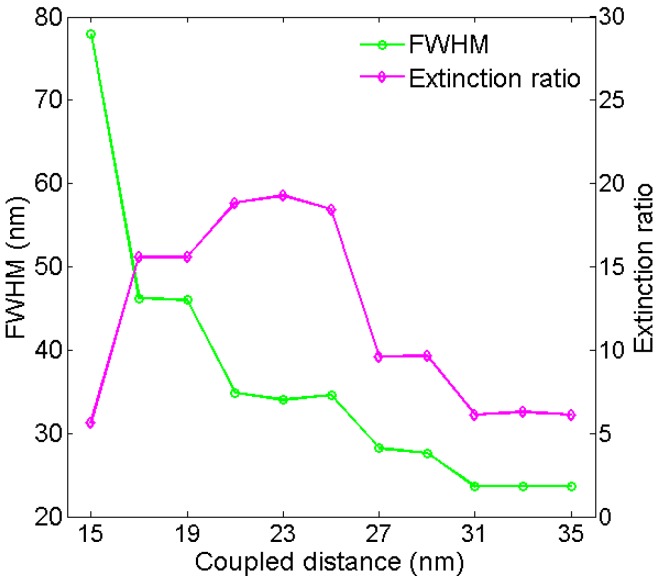
FWHM and extinction ratio as a function of coupling distance between waveguide and cavities for *L* = 320, *w* = 50 nm, and *n_d_* = 1.36048.

Moreover, based on the proposed structure, we investigate its temperature-sensing characteristics. The hexagonal cavities are sealed with ethanol with a high temperature coefficient of refractive index of 3.94 × 10^−4^ [43]. To find the suitable coupling distance for the plasmonic temperature-sensing structure, we study the influence of coupling distance on the FWHM and extinction ratio of its transmission spectrum. The coupling distance *d* is varied from 15 nm to 35 nm in increments of 2 nm, and other structural parameters are kept to be *w* = 50 nm, *L* = 320 nm, and *n_d_* = 1.36048 in order to make the resonance dip at the wavelength of near 1550 nm. In Figure 7, it plots the FWHM and extinction ratio of the resonance dip in the transmission spectrum at different coupling distances. As can be observed, the extinction ratio has an optimum value of about 19 dB at *d* = 23 nm, with a relatively better FWHM value of 34 nm. The FWHM value decreases to 23.6 nm after *d* = 31 nm, but the extinction ratio decreases to about 6 dB, which is too low and influences its practical application. Therefore, we choose *d* = 23 nm to construct our temperature-sensing structure.

**Figure 8:**
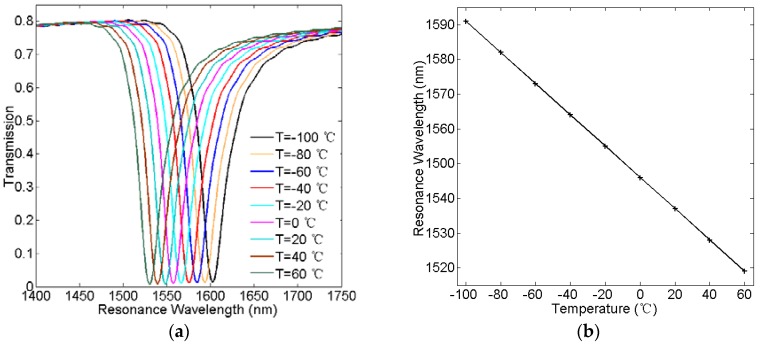
(**a**) Simulated transmission spectra of the temperature-sensing structure with different temperatures *T*; (**b**) Resonance wavelength versus the temperature *T*.

**Figure 9:**
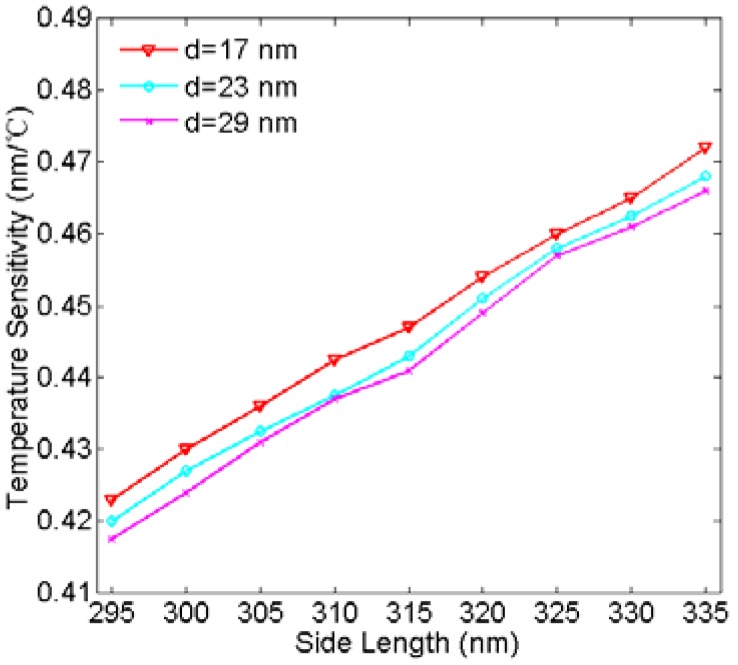
Temperature sensitivity versus side length for coupling distance *d* = 17 nm, 23 nm, and 29 nm, respectively.

Furthermore, we study the temperature sensitivity of the temperature-sensing structure. At first, to obtain the function between the resonance wavelength and the temperature, temperature of the ethanol medium is varied from −100 °C to 60 °C in steps of 20 °C, and others are set as *w* = 50 nm, *d* = 23 nm, and *L* = 320 nm, respectively. Figure 8a displays the simulated transmission spectra with different temperatures. As can be observed in Figure 8a, each resonance dip of the transmission spectra shows the same blue shift with the temperature increasing. Figure 8b plots the resonance wavelength as a function of the temperature. As shown in Figure 8b, it is found that the resonance wavelength has a linear relationship with the temperature. Therefore, we can understand the temperature by detecting the resonance wavelength. According to Equation (9), the corresponding temperature sensitivity is approximately 0.45 nm/°C. Next, to study the influence of side length *L* and coupling distance *d* on the temperature sensitivity, Figure 9 plots the temperature sensitivity as a function of the side length for *d* = 17 nm, 23 nm, and 29 nm. As can be observed in Figure 9, temperature sensitivity increases effectively as the side length increases. According to Equation (6), the increase of the side length makes the resonance wavelength of the cavity larger. Moreover, at the larger resonance wavelength, the change of the refractive index caused by the environment temperature has a greater impact on the change of the resonance wavelength. As a result, the temperature sensitivity will be increased. Additionally, the sensitivity slightly increases with the coupling distance increasing at the same side length. Therefore, the temperature sensitivity of the plasmonic temperature sensor can be optimized not only by tuning the side length of two hexagonal cavities but also the coupling distance.

**Figure 10:**
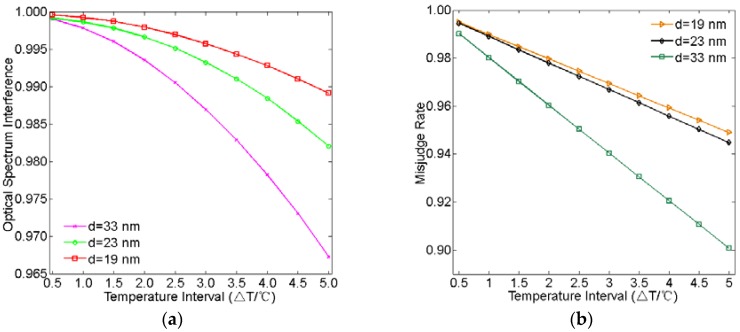
(**a**) Optical spectrum interference and (**b**) misjudge rate as a function of temperature interval for *d* = 33 nm, 23 nm, and 19 nm, respectively.

Finally, we use OSI and MR to analyze the temperature-sensing resolution of the plasmonic temperature sensor based on spectral interrogation. Figure 10a,b show OSI and MR as a function of the temperature interval for the coupling distance *d* = 33 nm, 23 nm, and 19 nm, respectively. As shown in Figure 10a,b, OSI and MR decrease as the temperature interval increases for each coupling distance. OSI and MR have the smallest value at *d* = 33 nm and the largest at *d* = 19 nm for the same temperature interval. Moreover, OSI and MR decrease fastest for *d* = 33 nm and decrease slowest for *d* = 19 nm. This is attributed to the decrease of the FWHM of the resonance dip, which is in accordance with the theory analysis based on Equations (10)–(12). As we know, a smaller OSI and MR are better for accurately detecting a tiny change in temperature. Consequently, the smaller the OSI and MR values are, the better the detection of small temperature changes is. Based on the results, we can obtain a smaller OSI and MR by decreasing the FWHM of the resonance dip. In addition, we can also utilize a sensing medium with a higher refractive index temperature coefficient, which results in a larger shift in the resonance dip for the same temperature interval.

## 4. Conclusions

In summary, this paper proposes and investigates a novel plasmonic temperature-sensing structure constructed by an MIM waveguide with dual symmetric coupled hexagonal cavities. The simulation results by using the FDTD method show that the FWHM of the resonance dip can be narrowed down by decreasing the coupling distance between the MIM waveguide and the cavities, and a maximum extinction ratio can be obtained at an appropriate position. The temperature-sensing characteristics are investigated based on the relationship between the refractive index of the dielectric material and the environment temperature. The corresponding temperature sensitivity with spectral interrogation can reach about 0.45 nm/°C with an FWHM of 34 nm and an extinction ratio of about 19 dB at *d* = 23 nm. Furthermore, OSI and MR are introduced to analyze the temperature-sensing resolution. Based on OSI and MR, the temperature-sensing resolution can be improved by reducing the FWHM of the resonance dip or using a sensing medium with a higher refractive index temperature coefficient. This work is helpful in designing nanoscale optical sensors with high temperature sensitivity and a high sensing resolution.
